# A novel role of ethephon in controlling the noxious weed *Ipomoea cairica* (Linn.) Sweet

**DOI:** 10.1038/srep11372

**Published:** 2015-06-18

**Authors:** Zhong-Yu Sun, Tai-Jie Zhang, Jin-Quan Su, Wah Soon Chow, Jia-Qin Liu, Li-Ling Chen, Wei-Hua Li, Shao-Lin Peng, Chang-Lian Peng

**Affiliations:** 1 Guangdong Provincial Key Laboratory of Biotechnology for Plant Development, Key Laboratory of Ecology and Environmental Science in Guangdong Higher Education, College of Life Sciences, South China Normal University, Guangzhou 510631, China; 2 Division of Plant Science, Research School of Biology, College of Medicine, Biology and Environment, The Australian National University, ACTON, Australian Capital Territory 2601, Australia; 3 State Key Laboratory of Bio-control, Sun Yat-Sen University, Guangzhou 510275, China

## Abstract

Several auxin herbicides, such as 2, 4-D and dicamba, have been used to eradicate an exotic invasive weed *Ipomoea cairica* in subtropical China, but restraining the re-explosion of this weed is still a challenge. Since ethylene is one of the major intermediate functioning products during the eradication process, we explored the possibility, mechanism and efficiency of using ethephon which can release ethylene to control *Ipomoea cairica*. The results of the pot experiment showed that 7.2 g /L ethephon could totally kill *Ipomoea cairica* including the stems and roots. The water culture experiment indicated that ethephon released an abundance of ethylene directly in leaves and caused increases in electrolyte leakage, 1-aminocyclopropane-1-carboxylic acid (ACC), abscisic acid (ABA) and H_2_O_2_ and decreases in chlorophyll content and photosynthetic activity, finally leading to the death of *Ipomoea cairica*. The field experiment showed that the theoretical effective concentration of ethephon for controlling *Ipomoea cairica* (weed control efficacy, WCE = 98%) was 4.06 g/L and the half inhibitory concentration (I_50_) was 0.56 g/L. More than 50% of the accompanying species were insensitive to the phytotoxicity of ethephon. Therefore, ethephon is an excellent alternative herbicide for controlling *Ipomoea cairica.*

*Ipomoea cairica* (Linn.) Sweet is a perennial vine belonging to convolvulaceae, now one of the most noxious invasive weeds in south China^1^. This weed is considered to be a typical plant killer that is often seen heavily covering other plants in scenic spots, parks and wild lands. Its characteristics, such as a climbing habit, rapid growth rate, and a strong capacity for vegetative propagation, contribute to its quickly-attained dominance in a community.

Chemical control is still a main method to control the spread of *Ipomoea cairica* in China, although it leaves some chemical residues in the environment. It has been reported that 2-methyl-4-chlorine and 2, 4-D butylate can be used to eliminate *Ipomoea cairica*^2^. Meanwhile, dicamba and fluroxypyr have also shown significant efficacies in controlling the weed^3^. Although these four herbicides mentioned above are used, retraining the re-explosion of *Ipomoea cairica* is still a challenge in south China. The excessive application of the above four herbicides also damaged soil and caused some environmental pollution.

It have been revealed that these four herbicides mentioned above shared the same mode of action of auxin herbicides, in which the biosynthesis of abscisic acid (ABA) and ethylene are two major steps^4^. Ethylene is a gaseous phytohormone that induces fruit ripening and tissue senescence, and is often substituted by ethephon. Ethephon (C_2_H_6_ClO_3_P) is a widely used ethylene-releasing plant regulator in agriculture. Ethephon’s mode of action acts via liberation of ethylene, which is absorbed by the plant and interferes in the growth process. It has been reported that ethephon has a role in inhibiting plant growth^5^, promoting stomatal opening^6^ and flowering^7^,^8^, inducing pollen sterility^9^, influencing biosynthesis of secondary metabolites^10^,^11^,^12^ and fruit ripening, improving fruit quality^13^,^14^,^15^,^16^,^17^, enhancing herbicide efficacy pretreatment^18^, eradicating pests^19^, depressing vesicular arbuscular mycorrhiza formation^20^ and nodulation^21^, and facilitating the harvest of fruits^22^,^23^. Some research also indicates that high concentrations of ethephon are phytotoxic and can cause damage to plants^24^,^25^.

The two-step mode of action of auxin herbicides (i.e., the biosynthesis of ethylene and ABA) raises the question whether ethylene or ABA can be used as an alternative herbicide to control the invasive species *Ipomoea cairica*? To our knowledge, little research has focused on this issue as yet. In this paper, considering the relatively low price and little residue in environment, we discuss the possibility, mechanism and efficiency of using ethephon to control *Ipomoea cairica*. Three experiments were designed, i.e., a pot experiment, a water culture experiment and a field experiment, to answer the following questions: (1) To what extent can ethephon be used as an alternative herbicide to control *Ipomoea cairica*? (2)What is the mechanism?

## Results

### Pot experiment

**Figure 1:**
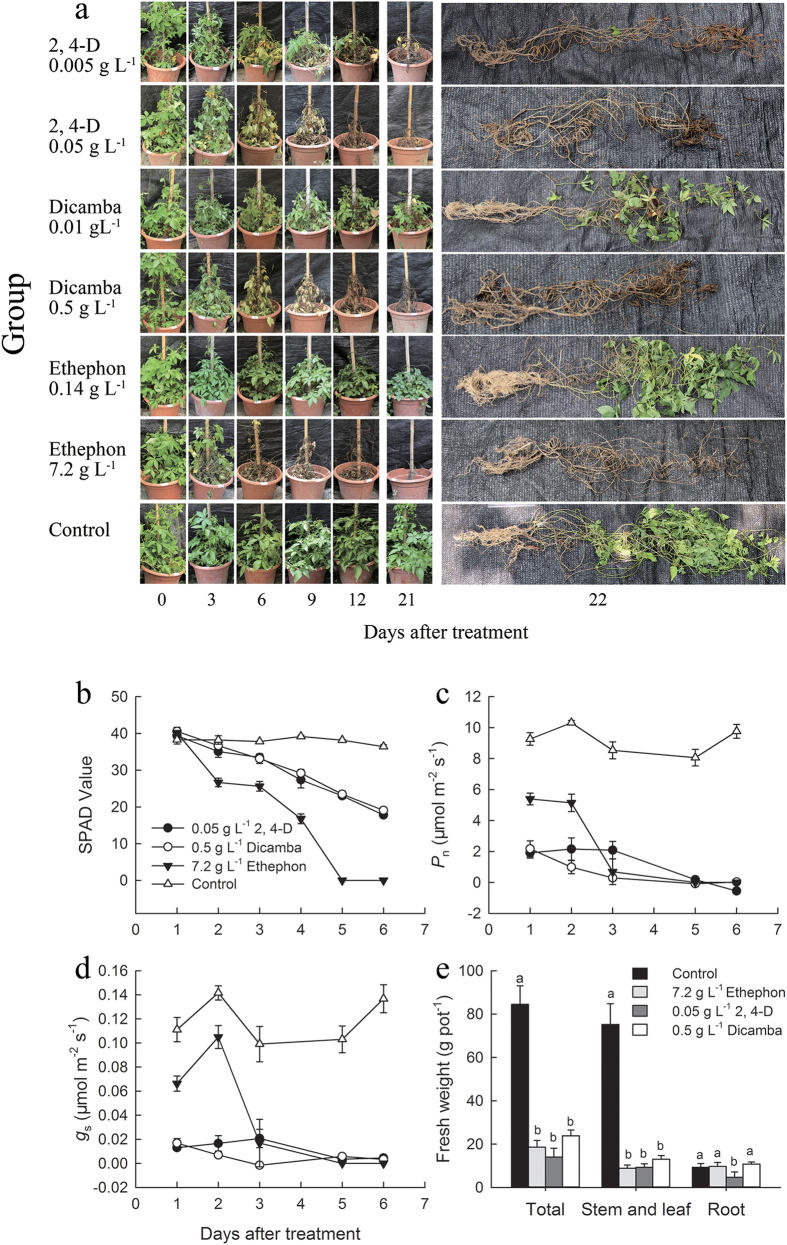
The change in phenotype (**a**), chlorophyll content (SPAD value, (**b**), net photosynthesis rate (Pn, **c**), stomatal conductivity (g_s_, **d**) and fresh weight (**e**) of *Ipomoea cairica* in a pot experiment. Data are means ± SE. The columns with different letters are significantly different with each other (*p* < 0.05).

The results showed that low concentrations of herbicides, namely, 0.005 g/L of 2, 4-D, 0.01 g/L of dicamba and 0.14 g/L of ethephon did not have obvious effect on controlling *Ipomoea cairica*. Many leaves were still alive after treatment for 21 days (Fig. 1a). Therefore, we will not discuss these three treatments in the following.

The other three treatments, i.e., 0.05 g/L of 2,4-D, 0.5 g/L dicamba and 7.2 g/L ethephon treatment, effectively controlled *Ipomoea cairica*, especially 7.2 g/L ethephon (Fig. 1a). The stems and leaves of *Ipomoea cairica* treated with 7.2 g/L ethephon were all dead after 6 days. Three days after treatments, most leaves of *Ipomoea cairica* in 7.2 g/L ethephon group withered and dropped, which did not happen in 2, 4-D and dicamba treatments at the same duration.

The total fresh weight, fresh weight of stem and leaf, and fresh weight of root were measured 22 days after treatments. They were all significantly different (*P* < 0.05) between each treatment and the control (Fig. 1e). The number of live leaf, the death rate of stem, and the death rate of root were also measured 21 days after treatments (Table 1). The results indicate that 0.05 g/L of 2, 4-D and 7.2 g*/*L of ethephon totally killed the stems, leaves and roots of *Ipomoea cairica*. On the other hand, 0.5 g/L dicamba was also able to entirely kill the stems and leaves of *Ipomoea cairica* but not the roots (Fig. 1a and Table 1).

Compared with 2, 4-D and dicamba, 7.2 g/L of ethephon injured *Ipomoea cairica* more quickly (in 3 days) and more effectively. The physiological responses of *Ipomoea cairica* to 2, 4-D, dicamba and ethephon were shown in Fig. 1. The chlorophyll content (SPAD value) declined sharply on the second day after treatment with 7.2 g/L of ethephon, faster than in the other two treatments. On the first day, net photosynthesis rate (Pn) in three treatments decreased by about one third or more relative to the control group; then all decreased to 0 within 3 to 5 days (Fig. 1c). The leaf stomatal conductance (g_s_) of *Ipomoea cairica* under 7.2 g/L ethephon treatment maintained higher than the other two treatments in the first 2 days, then decreased to almost zero on the third day together with the other two treatments (Fig. 1d).

## Water culture experiment

### Visual Change

**Figure 2:**
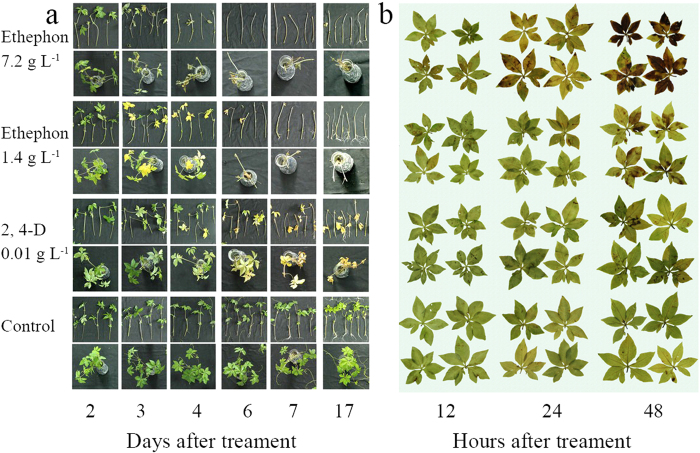
(**a**) The visual change of *Ipomoea cairica* after up to 17 days’ treatment in a water culture experiment; (**b**). The accumulation of H_2_O_2_ in leaves of *Ipomoea cairica* in a water culture experiment.

The results indicated that 1.4 g/L and 7.2 g/L ethephon quickly damaged all leaves of *Ipomoea cairica* in 3 days, while 0.01 g/L 2,4-D needed 6 to 7 days. Root growth of *Ipomoea cairica* was inhibited by 7.2 g/L ethephon which did not happen in other two treatments (Fig. 2a). After the treatments for 17 days, all leaves of *Ipomoea cairica* died and fell off the stems in 1.4 g/L and 7.2 g/L ethephon treatments, whilst there were still some yellow dead leaves attached to the stems in the treatment with 0.01 g/L 2, 4-D.

### Relative water content (RWC) and Electrolyte Leakage (EL)

**Figure 3:**
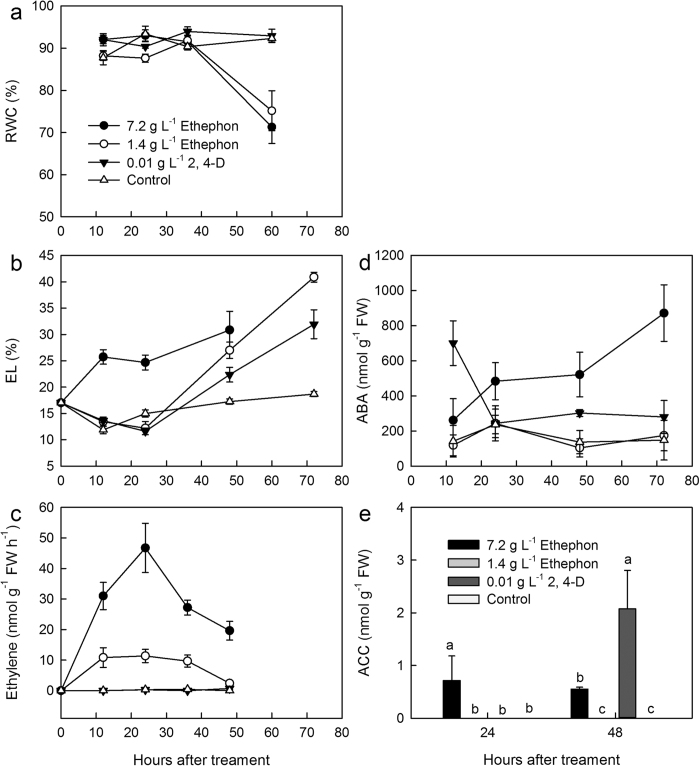
The change in RWC (**a**), EL (**b**), ethylene (**c**), ABA (**d**), and ACC (**e**) in leaves of *Ipomoea cairica* in the water culture experiment. Data were means ± SE. The columns with different letters are significantly different with each other (*p* < 0.05).

In treatments with 1.4 g/L and 7.2 g/L ethephon, the leaf RWC of *Ipomoea cairica* largely decreased after 60 h in contrast with the other treatments (Fig. 3a). The electrolyte leakage (EL) of *Ipomoea cairica* treated with 7.2 g/L ethephon increased to twice the level of the other groups after 12 h. In the other groups, EL decreased in first 24 h and then increased (Fig. 3b). The results showed that damage to cytomembranes occurred more quickly in 7.2 g/L ethophon treatment than in the other groups.

### Release of Ethylene

After treatment with 7.2 g/L ethephon, leaves of *Ipomoea cairica* released large quantities of ethylene for at least 48 h, the release rate peaking within 24 h (Fig. 3c). In contrast, little ethylene was detected after treatment with 0.01 g/L 2,4-D. At the same time, ACC, the precusor of ethylene, was also observed in the 7.2 g/L ethephon treatment group, whereas ACC was neither detected in the 0.01 g/L 2,4-D treatment group nor in the control group at hour 24 (Fig. 3e). After 48h treatment, ACC started to decrease in 7.2 g/L ethephon group, while its content increased greatly after treating with 0.01 g/L 2,4-D (Fig. 3e).

### Change in ABA and H_2_O_2_

Accompanying the release of ethylene and the accumulation of ACC, the content of ABA (Fig. 3d) and H_2_O_2_ also increased after 12 h in the 7.2 g/L ethephon treatment group (Fig. 2b). In 7.2 g/L ethephon treatment group, brown spots were obvious after 24h, and the intensity and area of the spots expanded quickly in the following 24 h (Fig. 2b). In contrast, no significant brown spots were observed after treatment with 0.01 g/L 2,4-D for 48 h.

## Field experiment

### Visual change

**Figure 4:**
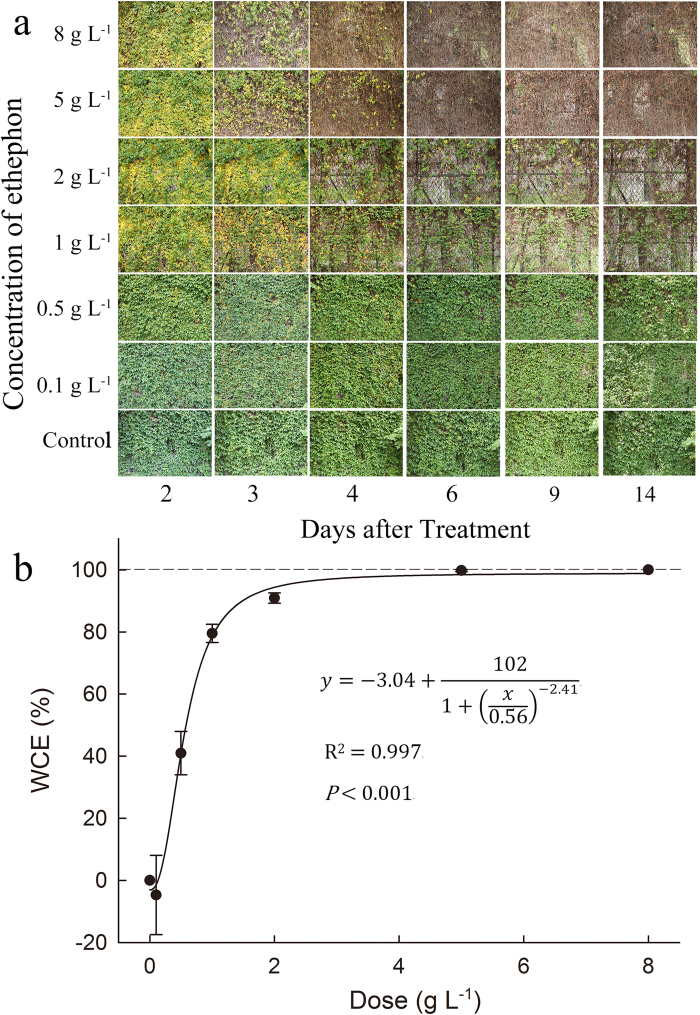
(**a**). The visual change of *Ipomoea cairica* treated with a concentration gradient of ethephon in a field experiment; (**b**). The logistic WCE-dose curve for the use of ethephon to control *Ipomoea cairica*. Data were means ± SE.

The results indicated that 0.1 g/L and 0.5 g/L ethephon did not have obvious effects on *Ipomoea cairica*. Few leaves turned yellow and fell off stems after 14 days’ treatment. When the concentration of ethephon increased to 1 g/L and 2 g/L, leaves of *Ipomoea cairica* turned yellow on the second day and began to fall on the third day (Fig. 4a); however , some leaves stayed on the stems after 14 days’ treatments. Almost all the leaves wilted and dropped off in 5 g/L and 8 g/L ethephon on the fourth day.

### The half inhibitory concentration and 98% effective concentration

The results indicated that WCE increased with the concentration of ethephon (Fig. 4b). WCE exceeded 90% when the concentration of ethephon was increased to 2 g/L. In 5 g/L and 8 g/L ethephon treatments, WCE reached to 99%–100%. The regression curve (Fig. 4b) demonstrated that the concentration for 98% effectiveness was 4.06 g/L , and I_50_ was 0.56 g/L for *Ipomoea cairica*.

### Effects of ethephon on accompanying species

There were 20 accompanying species, most of which were herbaceous species, including 5 other invasive species (*Ageratum conyzoides*, *Trilobate wedelia*, *Eupatorium catarium*, *Erigeron Canadensis*, and *Bidens bipinnata*), observed in our field experiment (Table 2). All three concentrations (5 g/L, 2 g/L and 1 g/L ) of ethephon in field experiment caused serious damage to *Ipomoea cairica*. The severity of phytotoxicity degrees reached levels 3–4, i.e., leaves withered or dead. Two herbaeous species, i.e., *Bidens bipinnata* (another invasive plant in south China) and *Mimosa pudica*, were also sensitive to ethephon. They suffered serious damage (Level 3) when treated with 1 g/L ethephon. There were also 3 herbaeous species, i.e., *Trilobate wedelia* (another invasive plant in south China), *Commelina communis*, and *Paeseria scandens*, which suffered serious damage (Level 3) under 5 g/L ethephon. On the other hand, ethephon had no effect (Level zero) or little effect (less than Level 2) on other accompanying trees and herbs.

## Discussion

There has been little research on the effect of ethephon on invasive weeds so far. Most previous research on ethephon has focused on its effects on the physiology of crops and economic trees, such as barley, apple, and olive^22^,^26^,^27^. Our pot experiment indicated that a high concentration of ethephon effectively eradicated *Ipomoea cairica*. As an herbicide, ethephon has the following advantages in controlling the invasive species *Ipomoea cairica*. (1) The response and action times are shorter. Most leaves of *Ipomoea cairica* withered and fell after treatment with 7.2 g/L ethephon for two days, and all leaves died after 6 days. In the other two treatment groups (0.05 g/L 2, 4-D and 0.50 g/L dicamba), some leaves of *Ipomoea cairica* withered on the sixth day but 9–12 days were needed for all leaves to die. (2) Ethephon leaves little chemical residue in the environment. Chemical residues of herbicides in agriculture can decrease the yield of subsequent crops. In natural succession, chemical residues may affect the establishment of later succession species. Ethephon decomposes into ethylene and the residue is volatile when pH > 4.0. Since the decomposition rate increases with temperature, ethephon can decompose fast in south China where the temperature can reach 40 °C in summer. By contrast, the residues of 2, 4-D and dicamba can last for several days to several months in soil^28^,^29^. (3) Ethephon can totally kill the roots of *Ipomoea cairica*, thereby preventing their re-growth. One of the biggest challenges in controlling *Ipomoea cairica* is controlling its re-proliferation. Previous research indicated that 2, 4-D and dicamba cannot totally kill the roots of *Ipomoea cairica*; the recovery growth happens when the treatments cease^17^.

Although the effective required concentration of ethephon is eight times the concentration of dicamba and 80 times that of 2, 4-D, due to its fast acting time, wide application in agriculture, commercial availability and relatively low price, ethephon is still an ideal alternative herbicide for controlling *Ipomoea cairica*.

Previous research indicates that a low concentration of ethephon can promote stomatal opening^6^ and a high concentration of ethephon can cause serious damage to guard cells^30^. After entering leaf tissues, ethephon releases a large amount of ethylene. Ethylene enhance the synthetic capability of RNA and promote the synthesis of proteins finally cause the formation of a separation layer and the abscission of organs^8^. In our research, as ethylene release increased, electrolyte leakage was also observed in leaves, indicating that cell membranes of leaves were damaged by substantial ethylene production. The stomatal conductivity of *Ipomoea cairica* in 7.2 g/L ethephon treatment was maintained at high values in first 2 days (Fig. 1), probably due to the damage of guard cells. The decreases in Pn of *Ipomoea cairica* in treatments with 0.5 g/L 2, 4-D or 0.05 g/L dicamba were mainly caused by the decrease in stomatal conductivity. On the other hand, in 7.2 g/L ethephon treatment, given the high stomatal conductivity in the first 2 days, the decrease in Pn might have been caused by impairment of carbon assimilation or disruption of the photosynthetic apparatus.

In water culture experiments, the rate of ethylene release increased sharply within 24 h, accompanied by increases in EL, ABA, and H_2_O_2_, while the decline of RWC, photosynthetic activity and loss of chlorophyll were more gradual. This indicates that a large amount of released ethylene damaged the cell membrane of leaves of *Ipomoea cairica*. Ethylene also promotes the synthesis of ABA (Fig. 3d) which causes the over production of H_2_O_2_ (Fig. 2b), and finally damages the tissues of *Ipomoea cairica*.

**Figure 5:**
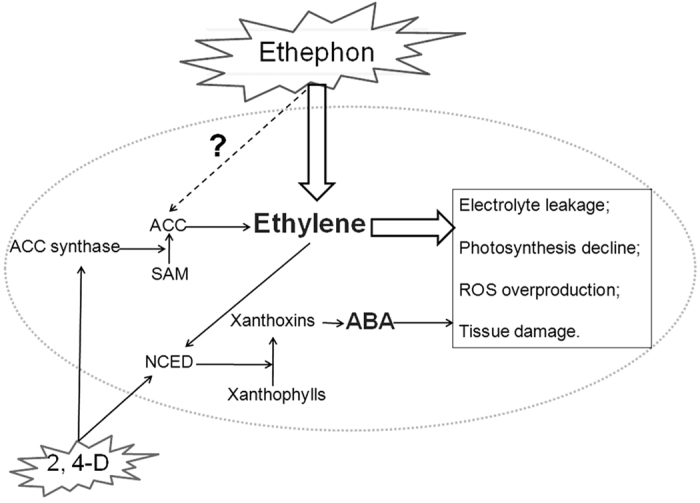
The difference in modes of action between ethephon and 2, 4-D. 2, 4-D induces the production of ACC synthase, therefore catalyzes the synthesis of ACC. ACC is the precursor of ethylene and can be converted into ethylene under catalysis by ACC oxidase. 2, 4-D also induces the production of NCED, which catalyzes the pyrolysis of xanthophylls to form xanthoxin, and finally turned to ABA. Ethylene and ABA cause the damage of cell membrane and the increase of RWC and H_2_O_2_ in plant tissues. In contrast with 2, 4-D, ethephon does not require ACC to produce ethylene and releases abundant ethylene directly after entering tissues. ACC: 1-aminocyclopropane-1-carboxylate; ABA: abscisic acid; NCED: 9-cis-epoxycarotenoid dioxygenase; SAM: S-adenosylmetionine.

The results of the water culture experiment demonstrate that the mechanism of using ethephon to control *Ipomoea cairica* is similar to that using other auxin herbicides. Ethylene and ABA are two major factors leading to damage of plant tissues after treatment with auxin herbicides^4^,^31^,^32^. Taking 2, 4-D as an example (see Fig. 5), after entering into *Ipomoea cairica*, 2, 4-D induces the production of ACC synthase, consequently catalyzing the synthesis of ACC^25^. ACC is the precursor of ethylene and can be converted into ethylene under the catalyzation of ACC oxidase. In addition, 2, 4-D also induces the production of 9-cis-epoxycarotenoid dioxygenase (NCED), which catalyzes the pyrolysis of xanthophylls to form xanthoxin, and finally ABA^32^. Ethylene and ABA lead to damage of cell membranes and the increase of RWC and H_2_O_2_ in plant tissues.

However, there is one major difference between ethephon and other auxin herbicides after they enter plant tissues. Unlike other auxin herbicides, ethephon can release abundant ethylene directly, without requiring the precursor ACC (Fig. 5). This may be the reason that damage to *Ipomoea cairica*, such as loss of chlorophyll and increase of EL, occurred faster in the treatment with ethephon.

Since the release of ethylene associated with ethephon does not need activation of the relevant genes of ACC synthase, there is less chance for the emergence of resistant weeds compared with the use of other auxin herbicides. By contrast, relatively low concentrations of 2, 4-D or dicamba can activate the relevant genes of ACC synthase. We have used a higher concentration of ethephon than 2, 4-D or dicamba to control *Ipomoea cairica*. If the release rate of ethylene could be improved and prolonged, the effective concentration of ethephon required to control *Ipomoea cairica* will decrease.

## Conclusions

Ethephon releases abundant ethylene and then promotes the production of ABA and the accumulation of H_2_O_2_, finally damaging the *Ipomoea cairica* tissues. Because there is no requirement for the ethylene precursor ACC, ethephon can quickly and totally kill the leaves, stems and roots of *Ipomoea cairica*, while having little effect on the accompanying species. In addition, ethephon is easily degraded, more economical and practical, and leaves little chemical residues. Theoretically, ethephon is an excellent alternative herbicide for controlling the invasive species *Ipomoea cairica*, one that may well prove to be superior in practice.

## Methods

### Pot experiment

A collection of stem segments (12 cm long) of *Ipomoea cairica* were planted in 24 plastic pots (diameter 30 cm and depth 25 cm) filled with the same rich organic soil. Every pot had 3 stem segments. After 8 months of cultivation under natural conditions, 21 pots were selected and evenly divided into seven groups. Three groups were treated with 300 mL of 1.4 g/L ethephon, 7.2 g/L ethephon and water (control group), respectively. The other four remaining groups were taken as contrasts and treated with 300 mL of 0.005 g/L 2,4-D, 0.05 g/L 2,4-D, 0.01 g/L dicamba, and 0.5 g/L dicamba, respectively. In the following one week, photosynthetic characteristics and relative content of chlorophyll were measured every day. The biomass was measured after 21 days treatments. The net photosynthetic rate (Pn), stomatal conductance (g_s_), and transpiration rate (Tr) were measured by Li 6400 (Li-Cor, USA). The light intensity was set at 800 μmol m^−2^·s^−1^. The relative content of chlorophyll was measured by SPAD-502 (Minolta, Japan).

### Water culture experiment

A number of stem segments (10 cm long) of *Ipomoea cairica*, each containing 5 to 6 leaves, were selectively collected from the wild. The collected stem segments were assigned to 15 conical flasks (4–7 stem segments each conical flask). These 15 conical flasks were evenly divided into 5 groups. The stem segments in each group were dipped in 0.01 g/L 2,4-D, 0.14 g/L, 1.4 g/L and 7.2 g/L ethephon, or water (control group) for 10 seconds respectively and then cultivated in laboratory with natural light and temperature for 72 hours. During this period, the relative water content (RWC), electrolyte leakage (EL), release rate of ethylene, content of ACC, ABA and H_2_O_2_ of leaves were monitored every 12 hours.

### Relative water (RWC)

Complete leaves were removed from each treated sample and the fresh weights (FW) were immediately measured. The leaves were then soaked in water for 8 hours and the turgid weight (TW) was recorded. Subsequently, the leaves were oven-dried to reach a constant dry weight (DW) at 80 °C. Relative water content (RWC) was calculated according to Downey and Miller^33^:
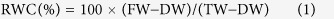


### Electrolyte leakage

Leaf from each treated sample was first washed with tap water and then with deionized water. Fifteen fresh leaf discs (0.75 cm diameter) from each sample were cut and put into a test tube with 15 mL deionized water. The electrolyte content in the solution was measured immediately (C_0_) and again (C_2_) after 10 min of vacuum using a conductivity meter (FE30, Mettler-Toledo Group, Switzerland). Total electrolyte (TC) was determined after 15 minutes of boiling in a water bath. Results were expressed as percentage of electrolyte leakage: EL (%) = 100 × (C_2_−C_0_)/(TC−C_0_)

### Rate of ethylene release

In order to measure the release rate of ethylene, 2 g of fresh leaf tissue from each treated sample was put in a gas-tight syringe (50 mL), respectively. Each syringe valve was moved to the 20 mL mark and the needle head was sealed with a small piece of rubber. After 1 h incubation, the gas was transferred to a reagent bottle. Finally, 1 mL of the gas sample was withdrawn and the content of ethylene was analyzed by a gas chromatograph (GC2010, Shimadzu, Japan).

### Content of ACC

ACC content was measured according to Nichols *et al.*^34^. First, a 2 g fresh leaf from each treated sample was frozen and powdered in liquid nitrogen, and then homogenized with 5 mL of 3% (v/v) freezing perchloric acid. The final volume of each sample was adjusted to 12 mL. After standing overnight at 4 °C, the homogenates were centrifuged at 11000 × *g* under 4 °C for 30 min. Subsequently, 5 mL of each supernatant was mixed with 5 mL of 10 mM mercuric chloride and 30 mL of deionized water in a small reagent bottle. The bottles were then sealed with a serum cap, and injected with 10 mL of an ice-cold mixture (2:1 v/v) of commercial bleach (5.25% NaOCl) and saturated NaOH, respectively. The bottles were shaken for 15 seconds, placed in an ice bath for 3 min and then shaken again for 15 seconds. 20 mL gas of each sample was withdrawn and stored in a sample vial. The gas was analyzed by GC (GC2010, Shimadzu, Japan) and the ethylene converted from ACC was measured, then the content of ACC was calculated based on the content of ethylene.

### Content of ABA

For measuring the content of ABA, 2 g fresh leaf from each treated sample was powdered in liquid nitrogen, and then homogenized in a 5 fold volume of 80% methanol. After overnight extraction at 4 °C, the samples were filtered by suction, evaporated to one third of its original volume at 32 °C, and extracted 3 times with a double volume of petroleum ether to remove chlorophyll, carotenoids and lipids^35^,^36^. The pH of the aqueous-methanol phase was then adjusted to 8.0 with 0.1M NaOH. In order to extract and minimize other interfering substances in the aqueous phase^37^, polyvinylpyrrolidone (0.5 g) was added in the aqueous-methanol phase and filtered by suction after shaking for a few seconds. The aqueous-methanol phase (pH 8.0) was then extracted 3 times with twice volume of ethyl acetate. The ethyl acetate phase was discarded and the pH of the aqueous-methanol phase was adjusted to 3.0 with 1 M HCl. The aqueous-methanol phase was then extracted 3 times with the same volume of ethyl acetate. The ethyl acetate phase was kept and evaporated to dry at 32 °C. The residue of each sample was dissolved in 2 mL of methanol, filtered (0.45 μm pore size) and stored in sample vials at 4 °C. The stored samples were analyzed by high performance liquid chromatography (LC-20A, Shimadzu, Kyoto).

### Content of H_2_O_2_

In situ localization of H_2_O_2_ was measured using the diaminobenzidine (DAB) staining method. H_2_O_2_ could oxidize DAB into a brown precipitate, and the content of H_2_O_2_ could be measured by the number and intensity of brown spots on leaves stained by DAB. Leaves from each treated sample were infiltrated in 0.5 mg mL^−1^ DAB-phosphate buffer (50 mM, pH 5.8) for histochemical detection. Subsequently, leaf samples were infiltrated under vacuum for 30 minutes. The leaf samples were then held at room temperature until a brown color became visible. Chlorophylls in leaves stained deep-brown was removed by boiling in an ethanol:glycerine (9:1, v/v) solution. Photographs of the stained leaf samples were taken with a digital camera^38^,^39^.

### Field experiment

Seven quadrats (2 m × 2 m each) on the wattled wall covered with *Ipomoea cairica* were established in the wild. All species grown in every 2 m × 2 m quadrat were identified and recorded. Then every quadrat was divided into four smaller 0.5 m × 0.5 m sub-quadrats. Subsequently, 1.5 L of 0.1, 0.2, 1, 2, 5 and 8 g/L ethephon was sprayed on six 2 m × 2 m quadrats, respectively. One 2 m × 2 m quadrat sprayed with the same volume of water was taken as control group. Three 0.5 m × 0.5 m sub-quadrats of each group were selected to measure the weed control efficacy (WCE) after 16 days. WCE was calculated by the following equation:
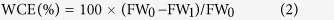


where FW_0_ is the fresh weight of *Ipomoea cairica* in the control group and FW_1_ is the fresh weight of *Ipomoea cairica* in a treatment group.

The half inhibitory concentration (I_50_) and 98% effective concentration were tested using logistic dose-WCE (weed control effect) curve^40^.

### Statistical Analysis

SAS system for Windows V8 was used to conduct one-way ANOVA analysis and nonlinear regression analysis.

## Additional Information

**How to cite this article**: Sun, Z.-Y. *et al.* A novel role of ethephon in controlling the noxious weed *Ipomoea cairica* (Linn.) Sweet. *Sci. Rep.* **5**, 11372; doi: 10.1038/srep11372 (2015).

## Figures and Tables

**Table 1:** The number of live leaves, death rate of stems and death rate of roots of *Ipomoea cairica* after three weeks of treatments.

**Treatment**	**Concentration (g/L)**	**Number of Live Leaf**	**Death rate of stem (%)**	**Death rate of root (%)**
2,4-D	0.05	0	100	100
Dicamba	0.5	0	100	61 ± 20
Ethephon	7.2	0	100	100
Control	**-**	108 ± 9	5 ± 5	0

**Table 2:** The phytotoxicity of ethephon on *Ipomoea cairica* and accompanying species.

**Family**	**Life form**	**Species**	**Degree of phytotoxicity under different concentrations of ethephon**		
			**5g/L**	**2g/L**	**1g/L**
Euphorbiaceae	Tree	*Bischofia javanica*	-	-	0
Moraceae	Tree	*Ficus hispida*	2	2,0	0
Ulmaceae	Tree	*Trema tomentosa*	-	0	-
Convolvulaceae	Herb	*Ipomoea cairica*	4,3	4,3	4,3
Araceae	Herb	*Colocasia antiquorum*	0	-	-
Araceae	Herb	*Alocasia macrorrhiza*	2	-	1
Araliaceae	Herb	*Schefflera arboricola*	-	-	0
Asteraceae	Herb	*Ageratum conyzoides*	-	2	-
Asteraceae	Herb	*Trilobate wedelia*	3	-	-
Asteraceae	Herb	*Eupatorium catarium*	-	2	-
Asteraceae	Herb	*Erigeron canadensis*	-	2	-
Asteraceae	Herb	*Bidens bipinnata*	3	3	3
Asteraceae	Herb	*Artemisia argyi*	-	-	0
Commelinaceae	Herb	*Commelina communis*	3	-	-
Mimosaceae	Herb	*Mimosa pudica*	-	-	3
Poaceae	Herb	*Digitaria ciliaris*	-	0	-
Poaceae	Herb	*Neyraudia reynaudiana*	1	-	0
Poaceae	Herb	*Oplismenus compositus*	-	0	-
Polygonaceae	Herb	*Polygonum chinensis*	0	0	-
Rubiaceae	Herb	*Paeseria scandens*	3	-	-
Urticaceae	Herb	*Boehmeria nivea*	0	-	-

Note: 0: no phytotoxicity; 1: little phytotoxicity, with malformed leaves; 2:middle phytotoxicity, with spots on leaves; 3:serious phytotoxicity, with withered leaves; 4:dead. The label “-” means the absence of species.
